# Determinants in the Underdiagnosis of COPD in Spain—CONOCEPOC Study

**DOI:** 10.3390/jcm11092670

**Published:** 2022-05-09

**Authors:** Myriam Calle Rubio, Juan Luis Rodríguez Hermosa, Marc Miravitlles, José Luis López-Campos

**Affiliations:** 1 Pulmonology Department, Research Institute of Hospital Clínico San Carlos (IdISSC), 28040 Madrid, Spain; mcal01@ucm.es; 2 Department of Medicine, School of Medicine, Universidad Complutense de Madrid, 28040 Madrid, Spain; 3 Pulmonology Department, Hospital Universitari Vall d’Hebron, Vall d’Hebron Institut de Recerca (VHIR), Vall d’Hebron Barcelona Hospital Campus, 08035 Barcelona, Spain; marcm@separ.es; 4 CIBER de Enfermedades Respiratorias (CIBERES), Instituto de Salud Carlos III, 28029 Madrid, Spain; lcampos@separ.es; 5 Respiratory Disease Medical-Surgical Unit, Instituto de Biomedicina de Sevilla (IBiS), Hospital Universitario Virgen del Rocío/Universidad de Sevilla, 41013 Sevilla, Spain

**Keywords:** chronic obstructive pulmonary disease (COPD), respiratory symptoms, spirometry, underdiagnosis, autonomous community, Spain

## Abstract

Factors such as seeking medical attention for respiratory symptoms and health professionals ordering spirometry come into play in the underdiagnosis of chronic obstructive pulmonary disease (COPD). The objective of this study was to analyze seeking medical attention and the use of spirometry in individuals with chronic respiratory symptoms and to compare these results with those obtained in the 2005 and 2011 surveys. Material and Methods: A cross-sectional, observational, epidemiological study was conducted via phone interview in December 2020 in Spain, with a representative sample from 17 autonomous communities. The study design was identical to that of the studies carried out in 2005 and 2011 to evaluate the changes that have occurred in seeking medical attention and performing spirometry in Spain, as well as the variability between autonomous communities. Results: From 89,601 phone contacts, a total of 6534 respondents were obtained. A total of 24.8% reported having some chronic respiratory symptom, and 17.9% reported a respiratory disease. Only 51.6% of those who had some chronic respiratory symptom had seen their doctor, which was less likely among current smokers (OR: 0.599, 95% CI: 0.467–0.769, *p* < 0.001) and those living in a rural setting (OR: 0.797, 95% CI: 0.651–0.975, *p* = 0.027). A total of 68.7% of the individuals who saw a doctor reported having undergone spirometry, most frequently males (OR: 1.535, 95% CI: 2.074–1.136, *p* < 0.005), former smokers (OR: 1.696, 95% CI: 2.407–1.195, *p* < 0.003), and those seen by a pulmonologist (OR: 6.151, 95% CI: 8.869–4.265, *p* < 0.001). With respect to the 2005 survey, more frequent use of spirometry has been observed (42.6 vs. 68.7%), without any change in seeking medical attention for respiratory symptoms. There is a clear variability according to the autonomous community (*p* < 0.05). Conclusions: Many individuals with chronic respiratory symptoms do not seek medical attention and although the use of spirometry has increased in the past 15 years, it is still an important area that needs improving in the primary care setting, especially among women. Both of these factors can be determinants in the underdiagnosis of COPD and its variability between autonomous communities.

## 1. Introduction

Chronic obstructive pulmonary disease (COPD) is a public health problem of primary importance due to its elevated prevalence and mortality [1,2], along with its elevated impact on economic resources [3], largely due to underdiagnosis. Said underdiagnosis causes the majority of patients to go undetected and reach advanced stages of the disease while receiving inadequate treatment or delaying actions to stop smoking [3,4].

Two studies carried out in Spain more than ten years apart, EPISCAN I [5] and II [6], concluded that there is significant underdiagnosis, between 73% and 74.7% in the Spanish population ages 40–80 in 2007 and 2019, respectively, with significant variability between autonomous communities.

There are multiple complex factors involved in this underdiagnosis. These include limited awareness in the population of the importance of seeking medical attention to assess respiratory symptoms and limited awareness of medical professionals on the use of spirometry to assess respiratory symptoms [3,4].

Studies conducted in Spain in 2005 [7] and 2011 [8] reveal that not all those who have chronic respiratory symptoms seek medical attention, and the use of spirometry is also limited. In the past 10 years, awareness campaigns have been carried out in the population, along with the implementation of training programs and initiatives to provide equipment to improve the practice of spirometry [3].

The objective of this study was to analyze certain aspects of respiratory care according to the autonomous community, such as seeking medical attention and the use of spirometry in individuals over the age of 40 with chronic respiratory symptoms and also to evaluate changes that have occurred in the past 15 years. This analysis will allow us to establish more efficient strategies to improve the diagnostic process for COPD in the different autonomous communities.

## 2. Materials and Methods

CONOCEPOC is a cross-sectional, observational, epidemiological study conducted between December 2020 and May 2021 following a design identical to that of the studies carried out in 2005 [7] and 2011 [8]. Inclusion criteria were men and women over the age of 40 who agreed to answer a questionnaire over the phone. Participation was voluntary, confidential and anonymous, randomly dialing landline phone numbers [9]. The survey was conducted over the phone in Spain’s 17 autonomous communities. Sampling was performed according to the following stratification criteria: sex, age according to decade (40–50, 51–60, 61–70, and >70 years), and rural (<10,000 inhabitants) or urban (≥10,000 inhabitants). Equally distributed quotas were obtained by age, sex, and place of residence within each of the 17 autonomous communities of Spain, requiring a total of 384 responses in each. This sample size allows identical precision of the samples by community in the population estimates, with an error of 5% and a power of 80% for the prevalence of different variables higher than 5%, so that the overall sample obtained is representative of each of the autonomous communities and is stratified by age group and habitat (rural/urban).

### 2.1. Data Collection

The respiratory symptoms and diagnosis questionnaire used for the survey was based on that of the European Coal and Steel Community (ECSC), a questionnaire that has been translated and validated in Spanish [10]. Additionally, questions about the use of spirometry and the perceived severity of other chronic diseases were included, identical to those used in the previous studies [7,8]. Perceived health and disease severity was evaluated on a scale from 0 to 10 points, with a higher score meaning better-perceived health or greater severity. The criteria for high risk of COPD were at least 55 years old, have a history of tobacco use (current or former smoker), and have some chronic respiratory symptoms. The complete questionnaire is provided in Supplementary Materials.

### 2.2. Statistical Analysis

In the descriptive analysis, qualitative variables are presented with their frequency distribution. Quantitative variables are summarized with their average and standard deviation (SD), and the quantitative variables that show an asymmetrical distribution are summarized with the median and interquartile range (IQR). Comparisons were made between study groups (according to respiratory symptoms, seeing a doctor, undergoing spirometry, and reporting having a respiratory disease) and the frequency of doctor visits and undergoing spirometry were compared to the survey results from 2005 [7] and 2011 [8]. The association between qualitative variables was evaluated using the chi-squared test or Fisher’s exact test. Student’s *t*-test was used for continuous variables in cases of variable normality; otherwise, non-parametric tests were used. Multinomial logistic regression was used to predict seeking medical attention and performing spirometry. All statistically significant variables in the univariate model were introduced into the model. In all hypothesis tests, the null hypothesis was rejected with a type I error or error of less than 0.05. All statistical comparisons with an error probability of less than 5% were considered significant. Data processing and analysis were carried out using IBM SPSS Statistics v21 software (IBM Corporation, Armonk, NY, USA).

## 3. Results

**Figure 1:**
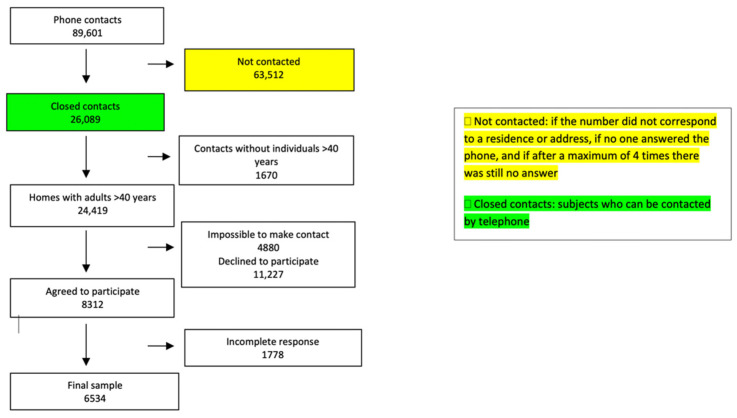
STROBE flowchart for the sample.

Of a total of 89,601 phone contacts, a final sample of 6534 respondents was obtained, with a response rate of 26.7%. The STROBE flowchart is presented in Figure 1. A total of 49.5% of the participants were male, and the average age was 61.5 years old. A total of 14.7% were current smokers, and 42.2% lived in a rural setting.

### 3.1. Chronic Respiratory Symptoms and Respiratory Disease Reported

A total of 1618 (24.8%) participants reported at least one chronic respiratory symptom. Of these, 627 (38.8%) met the criteria for high COPD risk. Shortness of breath was the most commonly reported symptom, present in 55.6%. Individuals who reported having chronic respiratory symptoms were older (63.8 ± 13.4 vs. 60.8 ±12.9, *p* < 0.001), had a greater tobacco exposure measured in pack-years (23.4 ± 19.4 vs. 16.5 ± 17.7, *p* = 0.001), and were more frequently current smokers (23.7% vs. 11.7%, *p* < 0.001) with a lower perceived health level (6.7 ± 1.9 vs. 7.6 ± 1.6, *p* < 0.001). There were no differences between sex or the rural/urban setting in the presence of referred chronic respiratory symptoms (Table 1).

A total of 1174 (17.9%) subjects reported having been diagnosed with a respiratory disease. Of these, 199 (3.1%) respondents reported having COPD. The individuals with respiratory disease were younger (60.5 ± 12.7 vs. 62.7 ± 14, *p* < 0.001), more frequently male (56% vs. 45.7%, *p* < 0.001), from a rural setting (47.3% vs. 42.4%, *p* = 0.007), current smokers (16% vs. 12.6%, <0.001), and had a lower perceived level of health (7.2 ± 1.8 vs. 7.4 ± 1.8, *p* = 0.001) (Table S1).

### 3.2. Seeking Medical Attention for Respiratory Symptoms. Associated Factors and Situation According to the Autonomous Community

**Figure 2:**
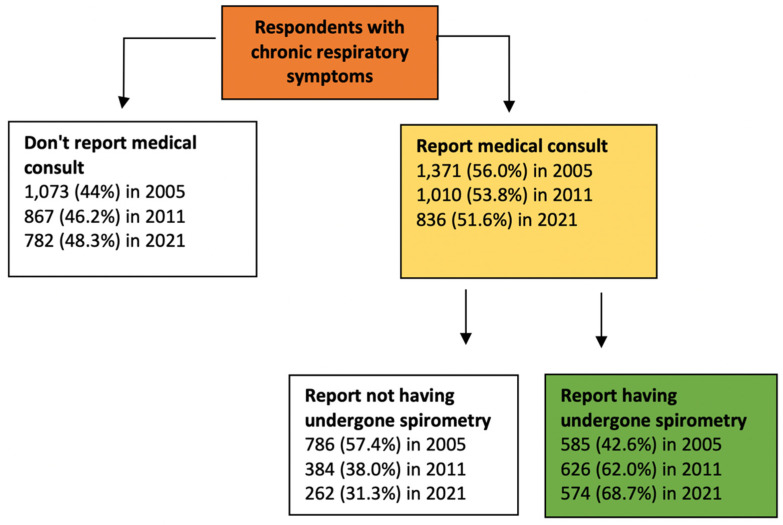
Changes in diagnostic flow of chronic respiratory symptoms in the Spanish population interviewed in 2005, 2011, and 2021.

A total of 836 (51.6%) of the subjects surveyed who had some chronic respiratory symptom had seen their doctor (56% saw their family doctor, and 44% saw a pulmonologist). The individuals who did not seek medical attention were younger (62.2 ± 13.3 vs. 65.2 ± 13.3, *p* < 0.001), more frequently lived in a rural setting (46.2% vs. 39.9%, *p* = 0.027), were more frequently current smokers (28.3% vs. 19.4%, *p* < 0.001), and had a higher perceived level of health (7 ± 1.7 vs. 6.36 ± 2.0, *p* < 0.001). There were no differences in sex or according to whether COPD risk criteria were met (Table 2). In the multivariate analysis, seeking medical attention was less likely among current smokers, residents in a rural setting, those without respiratory disease, and those who had never gone to the emergency room (Table 3). There were no significant differences found in the frequency of individuals with chronic respiratory symptoms who sought medical attention with respect to the previous studies in 2005 and 2011 (Figure 2). The analysis according to the autonomous community in the population surveyed in 2021 is shown in Figure S1.

### 3.3. Use of Spirometry. Associated Factors, Evolution over Time, and Situation According to the Autonomous Community

**Figure 3:**
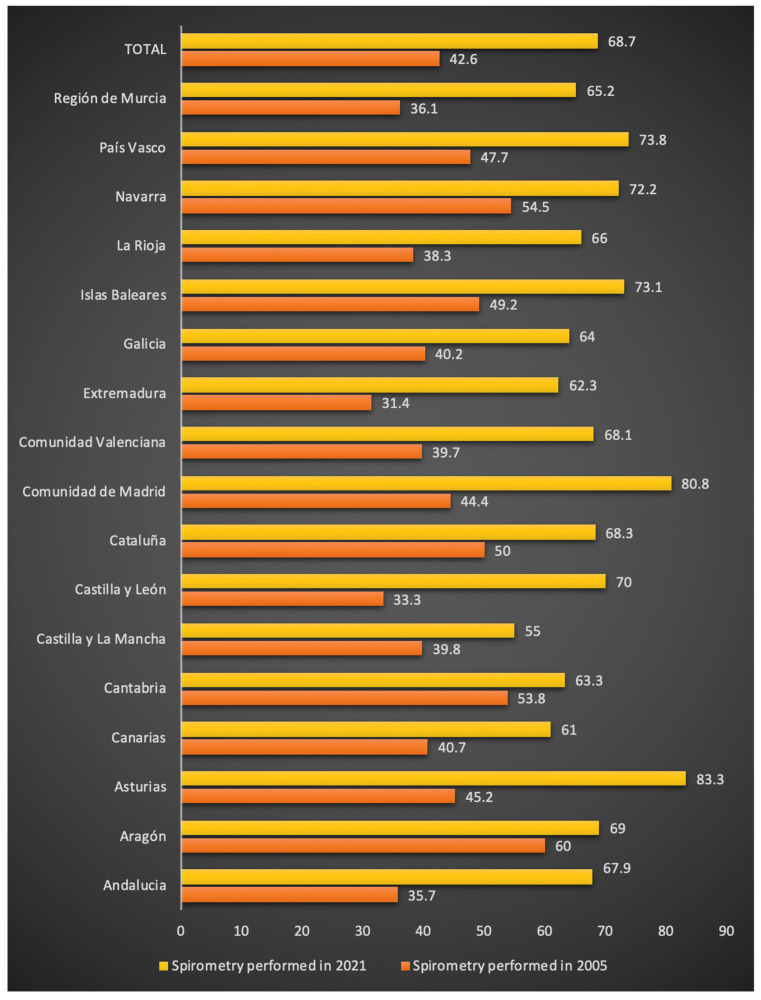
Use of spirometry in subjects with chronic respiratory symptoms in the Spanish population interviewed in 2005 and 2021. Data are expressed in frequencies (%).

Of those who reported respiratory symptoms and had seen a doctor, 574 (68.7%) said they had undergone spirometry. These individuals were more frequently younger (64.4 ± 13 vs. 66.8 ± 13.9, *p* = 0.018), lived in an urban setting (62.9% vs. 53.2%, *p* = 0.007), were male (53% vs. 42.7%, *p* = 0.006), had a history of smoking (55% vs. 43.9%, *p* = 0.008), met COPD risk criteria (46.5% vs. 25.1%, *p* < 0.001), and had gone to the emergency room for worsening of respiratory symptoms (25.6% vs. 10.3%, *p* < 0.001). The statistically significant variables in the univariate model are described in Table S2. In the multivariate analysis, the factors associated with a greater likelihood of performing spirometry were: being male, living in an urban setting, being a former smoker, being between 51 and 60 years old, meeting COPD risk criteria, being seen by a pulmonologist, and going to the emergency room for respiratory problems (Table 4). The magnitude and direction of the values were maintained after adjustment according to the autonomous community. Figure 2 shows the overall increase in the past 15 years, in 2005 (42.6%), in 2011 (62.0%), and in 2021 (68.7%). Figure 3 shows the changes compared to 2005 in the use of spirometry according to the autonomous community.

COPD risk: must be at least 55 years old, have a history of tobacco use (current or former smoker), and have some chronic respiratory symptom.

## 4. Discussion

The main results of this survey are that 24.8% of the adult population in Spain report suffering chronic respiratory symptoms. However, only 51.6% of those individuals have seen a doctor, and only 68.7% of them have undergone spirometry. Moreover, there was considerable variability in the use of spirometry and in seeking or accessing medical attention according to the autonomous community. This variability could help explain the variability in the underdiagnosis of COPD observed in the EPISCAN II population study [6].

In our analysis, chronic respiratory symptom frequency was similar to that found in previous studies in the Spanish adult population over the age of 40 following the same methodology, using the ECSC questionnaire [10] in 2005 (24%) [7] and the CONOCEPOC survey in 2011 (28.8%) [8]. Most studies that have evaluated changes in respiratory symptom prevalence over time show a rising trend in respiratory symptom prevalence rates [11,12]. However, a study conducted in Sweden [13] showed a reduction in cough and expectoration prevalence (from 12.4% to 10.1% and from 19.0% to 15.0%, respectively) over a 10-year period. These results could be related to different characteristics in the population studied and the changes that occur in risk factors for chronic respiratory disease over time, such as aging, smoking, atmospheric pollutants, occupational exposure [2], and obesity. These aspects have not been evaluated in our survey. In 2020, the combined prevalence of obesity and excess weight in the adult population in Spain was 53.6%, which shows an increase in the past 25 years [14]. This could explain the high frequency of dyspnea we recorded. In our analysis, patients with chronic respiratory symptoms were older and had a higher current tobacco index. Other factors to take into consideration were education and socioeconomic level. Previous studies have shown that subjects with lower levels of education are more than twice as likely to report chronic respiratory symptoms [15]. This aspect was not evaluated in our survey and could be biased in the population studied since the survey was conducted among landline phone users. The urban lifestyle has also been described as a risk factor for respiratory symptoms associated with allergic rhinitis and COPD [16]. However, in our study, we did not find any differences between the rural and urban settings in terms of respiratory symptom frequency.

In our survey, 17.9% of respondents reported having a respiratory disease. This frequency is similar to that reported in the study conducted in 2005 in Spain using the same methodology (reported by 20.9%) [7] and does not reflect the expected increase in respiratory disease due to demographic changes in the population pyramid or the increase in respiratory disease associated with obesity and insufficient control of toxic habits and environmental pollution. Currently, respiratory disease is the main reason for primary care visits (representing 20% of all visits) [17] and results in a significant number of hospitalizations, in addition to being a frequent cause of disability and lost life years and years of life with disability [2]. In our survey, a COPD diagnosis was only reported in 3.1% of cases, somewhat lower than that reported in 2005 (5.8%) [7] or in EPISCAN II [6] (5.3%).

The underdiagnosis of respiratory disease has been recognized in numerous epidemiological studies [5,6,18,19,20,21]. There are a variety of reasons for this, although it is largely due to the population’s limited perception of the need to seek medical attention for respiratory symptoms. In our survey, only half of the respondents (51.6%) reported seeing a doctor. This figure is similar to those found in 2005 (56%) [7] and 2011 (53.8%) [8], and it suggests that there have not been advances in the population’s perception regarding awareness and the importance of respiratory disease. However, the analysis according to the autonomous community shows a clear variability, with lower rates in Aragón (38.2%), Castilla-La Mancha (39.6%), and Extremadura (45.3%), while Catalonia (67.2%), Andalusia (64.4%), and Madrid (59.8%) show the highest rates of seeking medical attention. We must note that almost half of those meeting the criteria for high COPD risk (46.9%) reported not having seen a doctor. This figure shows a negative trend over the years compared to the rates reported in 2005, where 32.3% of individuals with a high risk had never seen a doctor. Factors associated with a lower likelihood of seeking medical attention were being a current smoker, living in a rural setting, not having gone to the emergency room, and not having a respiratory disease.

Another determinant of respiratory disease underdiagnosis, and specifically COPD, is healthcare professionals ordering spirometry. Only 68.7% of subjects who visited a doctor for symptoms reported having undergone spirometry. Although the frequency is greater than that reported in the studies conducted in Spain in 2005 (42.6%) [7] and in 2011 (62%) [8], it is still an area that needs to be improved. This is especially true among family doctors because more than 80% of the individuals who saw a doctor for respiratory symptoms without undergoing spirometry said they were seen in a primary care setting. This is a relevant figure since family doctors are patients’ first point of contact for possible COPD and the level of care at which early detection can occur. However, it should be pointed out that among the individuals considered to have a high COPD risk who sought medical attention, spirometry was performed in 80%. The factors associated with a greater likelihood of performing spirometry in individuals with respiratory symptoms who see a doctor were: being male, being between 51 and 60 years old, being a former smoker, meeting the criteria for high COPD risk or reporting having COPD, living in an urban setting, being seen by a pulmonologist, and having gone to the emergency room for worsening of symptoms. Our analysis confirms the persistent limited use of spirometry in women, and it suggests that physicians still have a low suspicion of COPD in women [22], which would explain the higher underdiagnosis in EPISCAN II due to the increased prevalence of COPD in women. This can be seen in the data in the EPISCAN II study [6], where the rate of underdiagnosis in Spanish women was 80.6%, 10 points higher than the 74.6% seen in men. The analysis of requested spirometry reported according to the autonomous community shows a clear variability, with a lower rate in Castilla-La Mancha (55%) and Extremadura (62.3%), while Asturias (83.3%), Madrid (80.8%), and the Basque Country (73.8%) show the highest reported rates of spirometry. However, when evaluating the change compared to 2005 in the use of spirometry, it must be noted that there is a greater increase in communities such as Castilla y León, Extremadura, and Madrid. However, it will be necessary to evaluate other aspects that may be involved in the diagnosis of COPD at the local level, such as educational campaigns [23], the protocolization of medical practice actions, equipment and space improvements, and periodic training to perform spirometry [24].

Our study is population-based and representative of the Spanish population, with more than 6000 responses in quotas from 384 participants in each of the 17 autonomous communities and has consistency in its results internally and with respect to the results of the surveys in 2011 and 2005. However, we must keep in mind that it was a phone interview with self-reported responses.

Although the random digit dialing sampling methodology is considered a gold standard despite its low yield (less than 25 percent in a nationwide sample) [9], and the response rate of 26.7% was higher than in previous surveys [7,8], it should be considered a limitation that could cause a bias in the population studied, since the landline telephone is used less in Spain due to the worsening of the economic crisis, only 77% of households in Spain have a landline telephone according to survey data from the National Institute of Statistics (INE) in 2019 [25].

## 5. Conclusions

Respiratory symptoms are highly prevalent in the general adult population. However, many individuals do not seek medical attention. This data has not improved in the past 15 years and makes informative actions to increase awareness regarding the relevance of respiratory disease among the population a priority. The use of spirometry has increased, but this area still requires improvement, especially in women who seek medical attention for respiratory symptoms and in the primary care setting. Both of these factors may be determinants in the underdiagnosis of COPD and its variability between autonomous communities.

## Figures and Tables

**Table 1:** Clinical and demographic characteristics of the surveyed population according to the presence of chronic respiratory symptoms.

	Total	With Respiratory Symptoms	Without Respiratory Symptoms	*p*-Value
N	6529	1618 (24.8)	4911 (75.2)	
Age (years), (average ± SD)	61.5 (13.1)	63.8 (13.4)	60.8 (12.9)	<0.001
Sex (male), n (%)	3238 (49.5)	796 (49.2)	2440 (49.7)	0.734
Setting < 10,000 inhabitants	2758 (42.2)	695 (45.6)	2063 (44.1)	0.302
Smoking history				
Accumulated consumption (pack-years), (average ± SD)	19.3 (18.7)	23.4 (19.4)	16.5 (17.7)	<0.001
Attempted to quit smoking, (average ± SD)	609 (63.6)	268 (70)	34.1 (59.3)	0.001
Attempts to quit, median (P25–P75)	2 (1–3)	2 (1–4)	2 (1–3)	0.94
Never smoked, n (%)	3554 (54.4)	736 (45.5)	2817 (57.4)	<0.001
Former smoker, n (%)	2018 (30.9)	499 (30.8)	1519 (30.9)	0.83
Current smoker, n (%)	958 (14.7)	383 (23.7)	575 (11.7)	<0.001
Have tried alternatives to tobacco, (%)	4.5	9.6	3.4	<0.001
Respiratory symptoms, (%)				
Chronic cough	17.5	450 (27.9)	-	
Chronic expectoration	9.5	325 (20.1)	-	
Chest wheezing or noises	12.9	269 (16.6)	-	
Shortness of breath or trouble breathing	55.6	1283 (79.3)	-	
COPD risk, n (%)		627 (38.8)		
Suffer from a respiratory disease, n (%)	1174 (17.9)	410 (25.3)	764 (15.5)	<0.001
Asthma	95 (1.5)	57 (3.5)	38 (0.7)	<0.001
Bronchitis or emphysema or COPD	199 (3.1)	125 (7.7)	74 (1.5)	<0.001
Perceived level of health *, (average ± SD)	7.4 (1.7)	6.7 (1.9)	7.6 (1.6)	<0.001
Self-reported health status, n (%)				<0.001
Poor (<5)	334 (5.1)	162 (10)	172 (3.5)	
Average (5–7)	2536 (38.9)	847 (52.3)	1690 (34.4)	
Good (>7)	3659 (56)	609 (37.5)	3049 (62.1)	

Note: Data expressed as mean (standard deviation) or in absolute (relative) frequencies according to the nature of the variable. The variable “attempts to quit smoking” was calculated only in the subgroup of current smokers. COPD risk: must be at least 55 years old, have a history of tobacco use (current or former smoker), and have some chronic respiratory symptom. * Perceived health was evaluated on a scale from 0 to 10 points, with a higher score meaning better perceived health.

**Table 2:** Characteristics of patients with respiratory symptoms according to medical attention sought.

	Patient with Chronic Respiratory Symptoms	Sought Medical Attention	Didn’t Seek Medical Attention	*p*-Value
N	1618	836 (51.7)	782 (48.3)	
Sex (male), n (%)	796 (49.2)	416 (49.8)	380 (48.6)	0.639
Age (years), (average ± SD)	63.8 (13.4)	65.2 (13.3)	62.2 (13.3)	<0.001
Setting < 10,000 inhabitants	695 (42.9)	334 (39.9)	361 (46.2)	0.027
Smoking history				<0.001
Current smoker, n (%)	736 (45.5)	162 (19.4)	221 (28.3)	
Former smoker, n (%)	499 (30.8)	269 (32.2)	230 (29.4)	
Never smoked, n (%)	383 (23.7)	405 (48.4)	331 (42.3)	
Chronic cough, (%)	27.9	27.1	29.7	0.501
Chronic expectoration, (%)	20.1	21.3	20.1	0.207
Chest wheezing or noises, (%)	16.6	16.4	16.4	0.976
Shortness of breath or trouble breathing, (%)	79.3	82.2	76.9	0.130
COPD risk, n (%)	627 (38.8)	333 (39.8)	294 (37.6)	0.356
Presence of respiratory disease reported, n (%)	410 (25.3)	227 (27.2)	183 (23.4)	0.009
Have gone to the emergency room for worsening of respiratory symptoms, n (%)	209 (12.9)	174 (20.8)	35 (4.5)	<0.001
Perceived level of health, (average ± SD)	6.7 (1.9)	6.3 (2.0)	7.0 (1.7)	<0.001
Doctor seen, n (%)				
Family medicine specialist		468 (56)		
Pulmonologist		368 (44)		

Data expressed as mean (standard deviation) or in absolute (relative) frequencies according to the nature of the variable. COPD risk: must be at least 55 years old, have a history of tobacco use (current or former smoker), and have some chronic respiratory symptom.

**Table 3:** Factors associated with seeking medical attention among those interviewed with respiratory symptoms. Multivariate analysis.

	OR (95% CI)	*p*-Value
Area of residence		
Area ≥ 10,000 inhabitants (ref.)		
Area < 10,000 inhabitants	0.797 (0.651–0.975)	0.027
Smoking status		
Never (ref.)		
Former smoker	0.956 (0.761–1.201)	0.698
Active smoker	0.599 (0.467–0.769)	<0.001
Presence of respiratory disease		
No (ref.)		
Yes	1.409 (1.090–1.822)	0.009
Have gone to emergency room for respiratory problem		
No (ref.)		
Yes	5.610 (3.845–8.186)	<0.001

**Table 4:** Factors associated with the use of spirometry among subjects with respiratory symptoms who visit a doctor. Multivariate analysis.

	OR (95% CI)	*p*-Value	Adjusted Autonomous Community OR (95% CI)	*p*-Value
Sex, female (ref.)				
Male	1.508 (2.024–1.123)	0.006	1.535 (2.074–1.136)	0.005
Area ≥ 10,000 inhab. (ref.)				
Area < 10,000 inhab.	0.655 (0.890–0.483)	0.007	0.691 (0.957–0.498)	0.026
Age 40–50 years old (ref.)				
51–60 years old	1.868 (3.112–1.121)	0.017	1.865 (3.136–1.109)	0.019
61–70 years old	1.068 (1.661–0.687)	0.769	1.135 (1.780–0.723)	0.582
>71 years old	0.910 (1.381–0.600)	0.659	0.902 (1.380–0.589)	0.633
Smoking status				
Never (ref.)				
Former smoker	1.684 (2.370–1.197)	0.003	1.696 (2.407–1.195)	0.003
Current smoker	1.394 (2.070–0.939)	0.099	1.393 (2.084–0.931)	0.107
COPD, No (ref.)				
Yes COPD	3.134 (13.746–0.715)	0.13	2.524 (11.564–0.551)	0.233
COPD risk	1.472 (1.998–1.085)	0.013	1.488 (2.035–1.088)	0.013
Doctor seen,				
Family (ref.)				
Pulmonologist	5.438 (7.728–3.826)	<0.001	6.151 (8.869–4.265)	<0.001
Emergency room for respiratory problem				
Yes (ref.)				
No	0.334 (0.518–0.215)	<0.001	0.335 (0.523–0.214)	<0.001

## Data Availability

The data presented in this study are available on request from the corresponding author.

## Supplementary Materials

The following supporting information can be downloaded at: https://www.mdpi.com/article/10.3390/jcm11092670/s1, Figure S1: Diagnostic flowchart for subjects interviewed in 2021 with chronic respiratory symptoms according to the autonomous community; Table S1: Clinical and demographic characteristics of the surveyed population according to the presence of respiratory disease; Table S2: Clinical and demographic characteristics of the surveyed population with symptoms seeking medical attention according to the use of spirometry.
