# Supplementary material for: Determinants in the Underdiagnosis of COPD in Spain—CONOCEPOC Study
Source: J Clin Med. 2022 May 9;11(9):2670. doi: 10.3390/jcm11092670 (PMC9105961; doi:10.3390/jcm11092670)
Supplement: Supplementary file 1 [file jcm-11-02670-s001.zip › jcm-1657318-supplementary.pdf]

Supplementary Materials

Supplementary Table S1. Clinical and demographic characteristics of the surveyed population according to the presence of respiratory disease.

N= 4206	With respiratory disease	Without respiratory disease	p-value
No. (%)	1174 (27.9)	3032 (72.0)	
Age (years), (average ± SD)	60.5 (12.7)	62.7 (14.0)	<0.001
Sex (male), n (%)	657 (56)	1385 (45.7)	<0.001
Setting <10,000 inhabitants	556 (47.3)	1286 (42.4)	0.007
Smoking history<br>Never smoked, n (%)<br>Former smoker, n (%)<br>Current smoker, n (%)	 613 (52.2)<br>373 (31.8)<br>188 (16)	 1824 (60.2)<br>827 (27.3)<br>381 (12.6)	<0.001
Tried to quit smoking, (%)	64.9	58.3	0.128
Perceived level of health*, (average ± SD)	7.2 (1.8)	7.4 (1.8)	0.001
Self-reported health status, n (%)<br>Poor (<5)<br>Average (5-7)<br>Good (>7)	 73 (6.2)<br>509 (43.4)<br>592 (50.4)	 162 (5.3)<br>1145 (37.8)<br>1725 (56.9)	0.001
Suffer from a respiratory disease, n (%)<br>Asthma<br>Bronchitis or emphysema or COPD	 95 (8.1)<br>199 (16.9)	-	
Have undergone spirometry, n (%)	159 (70)	144 (56)	0.001

Note: Data expressed as mean (standard deviation) or in absolute (relative) frequencies according to the nature of the variable.
The variable “tried to quit smoking” was calculated only in the subgroup of current smokers.

Supplementary Table S2. Clinical and demographic characteristics of the surveyed population with symptoms seeking medical attention according to the use of spirometry.

	Spirometry performed	Spirometry never performed	p-value
N (%)	574 (68.7)	262 (31.3)	
Sex (male), n (%)	304 (53)	112 (42.7)	0.006
Age (years), (average ± SD)	64.4 (13.0)	66.8 (13.9)	0.018
Setting <10,000 inhabitants	213 (37.1)	121 (46.1)	0.007
Smoking history<br>Current smoker, n (%)<br>Former smoker, n (%)<br>Never smoked, n (%)	 115 (20)<br>201 (35)<br>258 (44.9)	 47 (17.9)<br>68 (26)<br>147 (56.1)	0.008
COPD risk, n (%)	267 (46.5)	66 (25.1)	<0.001
Shortness of breath reported, (%)<br>Chronic cough, (%)<br>Chronic expectoration, (%)<br>Chest wheezing or noises, (%)	82.1<br>27<br>21.5<br>17.5	82.5<br>28.1<br>20.8<br>15.8	0.954<br>0.685<br>0.165<br>0.547
Presence of respiratory disease reported, n (%)	159 (52.5)	68 (37.6)	0.001
Have gone to the emergency room for worsening of respiratory symptoms, n (%)	147 (25.6)	27 (10.3)	<0.001
Doctor seen, n (%)<br>Family medicine specialist<br>Pulmonologist	 255 (44.4)<br>319 (55.6)	 213 (81.3)<br>49 (18.7)	<0.001
Perceived level of health, (average ± SD)	6.2 (2.0)	6.5 (2.0)	0.153

Data expressed as mean (standard deviation) or in absolute (relative) frequencies according to the nature of the variable.
COPD risk: must be at least 55 years old, have a history of tobacco use (current or former smoker) and have some chronic respiratory symptom.

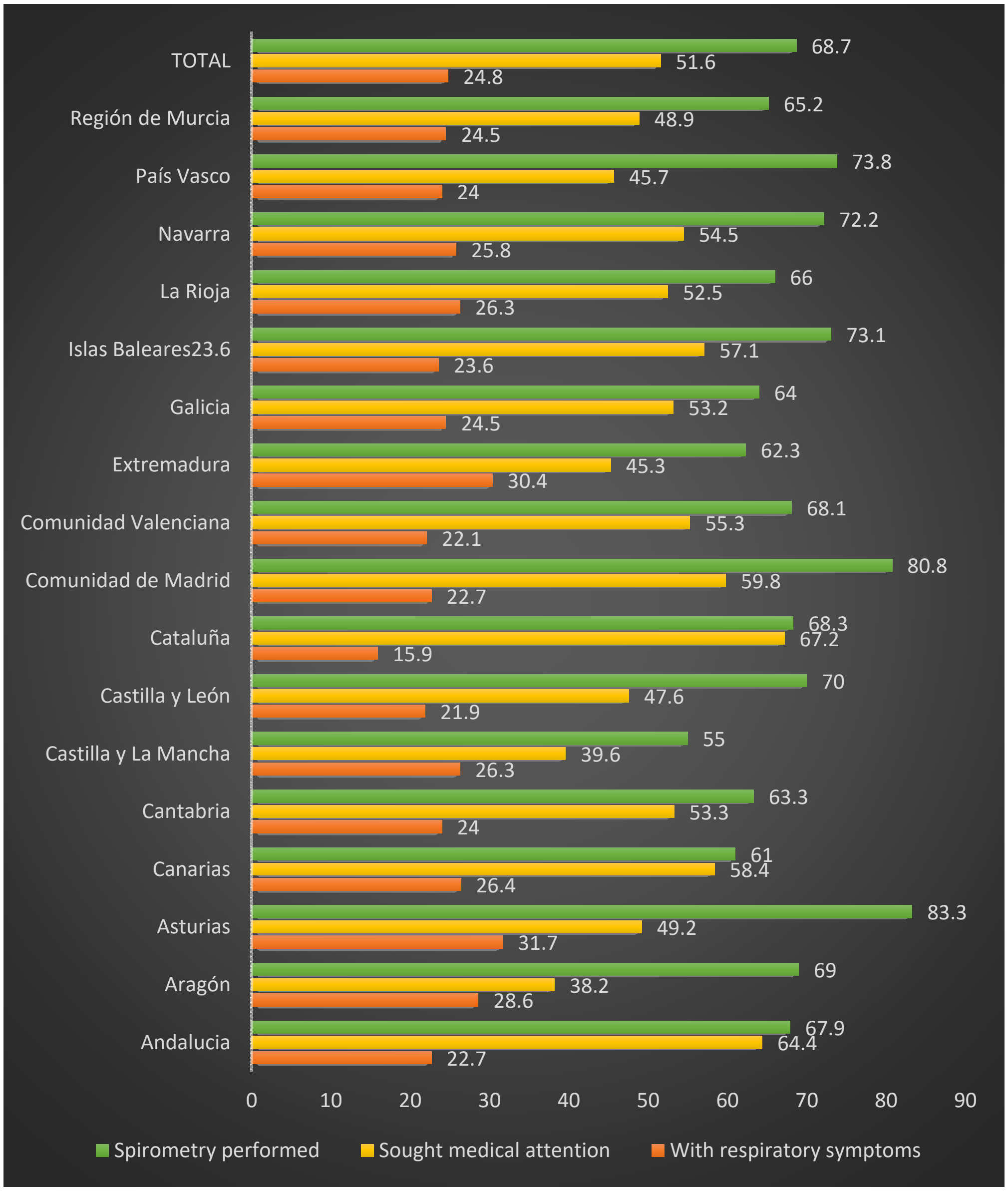

Supplementary Figure S1. Diagnostic flowchart for subjects interviewed in 2021 with chronic respiratory symptoms according to autonomous community. Data expressed in frequencies (%).

CONOCEPOC

QUESTIONNAIRE

Hello, I'm ………………….

We're conducting a study sponsored by the Spanish Pulmonology Society to research spirometry awareness. Participation in this study is voluntary and all data collected will be anonymous. The only drawback for you would be taking the time to answer a few short questions, which won't take more than 10 minutes.

Do you agree to participate and consent to your answers being recorded in order to analyze them anonymously?

Thank you very much.

SOCIODEMOGRAPHIC DATA

S1 What is your age? _________

< 40 ☐ years old → ***End interview.***

☐ 40-50 years old

☐ 51-60 years old

☐ 61-70 years old

>70 ☐ years old

S2.- Sex *(make note without asking based on name, if possible)*:

☐ Male

☐ Female

S3.- Autonomous community*:

- ☐ Andalusia
- ☐ Aragon
- ☐ Asturias
- ☐ Madrid
- ☐ Canary Islands
- ☐ Cantabria
- ☐ Castilla-La Mancha
- ☐ Castilla y León
- ☐ Catalonia
- ☐ Extremadura
- ☐ Galicia
- ☐ Balearic Islands
- ☐ La Rioja
- ☐ Navarra
- ☐ Basque Country
- ☐ Murcia
- ☐ Valencia

S4.- Setting*:

- ☐ Up to 10,000 inhabitants
- ☐ over 10,000 inhabitants

<u>COPD AWARENESS - DIAGNOSIS</u>

C1.- On a scale from 0 (very poor) to 10 (very good), please indicate what you consider your general health status to be. |__|__|

C2.- Do you currently suffer from a respiratory disease?

- ☐ Yes → *Go to C3.*
- ☐ No → *Go to C4.*
- ☐ Don't know / No answer → *Go to C4.*

---

* This information is available in the phone number database for the general population.

Spontaneous knowledge

C3.- Please indicate which disease(s). *(**Don't** read options)*

- □ COPD (or chronic obstructive pulmonary disease)
- □ Asthma
- □ Chronic bronchitis
- □ Emphysema
- □ Other (indicate which other respiratory disease)

*If they **don't** indicate COPD, chronic bronchitis or emphysema in C3, answer C4.*

If they indicate COPD in C3, go to C5.

C4.- Do you know what COPD (chronic obstructive pulmonary disease) is? (record spontaneous knowledge)

□ Yes What are the main symptoms? (don't read)

□ Morning cough □ Wheezing while breathing

□ Expectoration/Sputum/Phlegm □ Shortness of breath □ Other

If they answer YES for C4 (go to C6)

No (go to C5)

C5. (Read) As you know, COPD is the name for chronic obstructive pulmonary disease, which encompasses a group of diseases like chronic bronchitis and emphysema, and which is characterized by a feeling of shortness of breath, cough, wheezing while breathing and fatigue resulting from smoking and other causes.

Does it sound familiar now? □ No (go to C7) □ Yes (go to C6)

C6.- How did you learn about it?

- □ Media (newspaper, radio, TV)
- □ Internet or social media
- □ Doctors
- □ Pharmacist
- □ Relative or acquaintance with disease

*C7.- Have you ever been diagnosed with the following diseases? Ask **only** about those **not** mentioned in C3.*

	Yes	No	Don't know
COPD	□	□	□
Asthma	□	□	□
Chronic bronchitis	□	□	□
Emphysema	□	□	□

*If COPD, chronic bronchitis or emphysema is **not** mentioned in C7 or in C2, go to E1.*

<u>COPD TREATMENT</u> *(Only if **COPD or chronic bronchitis or emphysema** is mentioned in C2 or C7)*

T1.- Do you follow a treatment plan for COPD?

- No
- Yes →T2. Please specify treatment (*read options*):
  - Inhalers
  - Medication
  - Oxygen
  - Support treatment to stop smoking

If inhalers are indicated in T2 (go to T3).

T3. On a scale from 0 (not at all difficult) to 10 (very difficult), please indicate your opinion of your inhaled treatment compliance.

Subject CHARACTERISTICS (for all respondents)

P1.- Do you smoke?

□ Yes, smoker P.1.1.1.- How many cigarettes do you smoke a day?

____

P.1.1.2.- How many years have you smoked?

____

P.1.1.3.- Have you ever tried to quit smoking?

□ Yes → P1.1.4.- How many times?

____

□ No

□ No, former smoker

P 1.2.1 How long ago did you stop smoking?

P.1.2.2.- How many cigarettes did you smoke a day? ________

P.1.2.3.- How many years did you smoke?

____

□ No, never smoked

P2 Have you ever tried alternatives to cigarettes?

□ Yes → Specify *(read options)*:

- E-cigarettes
- IQOS
- JUUL
- Other

□ No

P.3 Please indicate your attitude towards these alternatives compared to traditional cigarettes.

□Highly favorable

□Favorable

□Don't know

□Unfavorable

□Highly unfavorable

P.4 Do you think they can help to stop smoking? (in regard to these alternatives to traditional cigarettes)

□ YES

□ NO

□Don't know

P.5 Do you feel there are health risks?

(in regard to these alternatives to traditional cigarettes)

□ YES

□ NO

□Don't know

P6.- Have you had a more or less constant morning cough for more than three months a year for at least 2 years?

□ Yes □ No

P7.- Have you had more or less constant expectoration (cough with sputum) for more than three months a year for at least 2 years?

□ Yes □ No

P8.- Do you have more or less constant wheezing or noises while breathing for more than three months a year?

□ Yes □ No

P9.- Do you feel short of breath (trouble taking air in more than exhaling it)? *(Read options)*

□ No

□ When climbing a hill or two floors

□ When climbing one floor

□ When walking on a flat surface

□ At rest

If any of the following symptoms are indicated: Yes for P6, P7, P8, or ***not*** *indicating No for P9 (go to P10 and to P11)*

P10.- Have you seen a doctor for these problems? *(if necessary, reference cough, cough with sputum and/or shortness of breath, according to answer)*

□ No

□ Yes (specify):

Who?

□ primary care doctor

□ lung specialist/pulmonologist

Have you ever undergone spirometry (blowing into a device)?

□ No □ Yes

P11.- Have you ever gone to the emergency room for worsening of these respiratory problems? *(if necessary, reference cough, cough with sputum and/or shortness of breath, according to answer)*

□ No

□ Yes (specify how many times in the past year):

<u>COPD PERCEPTION (for all respondents)</u>

F1.- On a scale from 0 (not serious) to 10 (maximum severity), please indicate how serious you believe COPD to be. _________

F2.- On a scale from 0 (not serious) to 10 (maximum severity), please indicate how serious you believe the following illnesses to be:

Diabetes…………….

Hypertension……….

Angina pectoris…

Stomach ulcer

Arthrosis-arthritis………

Thank you very much for your participation.
